# Identification of Individual Texas Horned Lizards ( *Phrynosoma cornutum* ) Using Genotypes and Ventral Spot Patterns

**DOI:** 10.1002/ece3.71167

**Published:** 2025-03-24

**Authors:** Daniella Biffi, Mary R. Tucker, Alexis Ackel, Dean A. Williams

**Affiliations:** ^1^ Andrews Institute for Research in Mathematics & Science Education Texas Christian University Fort Worth Texas USA; ^2^ Department of Biology Texas Christian University Fort Worth Texas USA

**Keywords:** individual identification, microsatellites, natural markings, probability of identity

## Abstract

Identifying individuals within a species is vital for monitoring population dynamics and determining appropriate conservation efforts. Traditional methods for marking individual lizards include toe‐clipping, branding, tattooing, and passive integrated transponder (PIT) tags. However, some of these methods can potentially cause stress, affect performance and survival, and raise concerns about the ethical treatment of animals. We conducted a long‐term study on the urban ecology of Texas horned lizards living in two small towns in south Texas, USA. Our study was in the unique position of possessing a dataset of individuals that were PIT tagged, genotyped, and photographed, which allowed us to validate genotyping and natural markings for individual identification. We calculated our genotyping error rate by comparing genotypes of recaptured individuals identified by PIT tags. Our mean error rate per allele was 0.0016, our mean error rate per multilocus genotype was 0.032, and we had high power to identify individuals. We used HotSpotter software to match photographs of individuals identified by PIT tags and genotyping. HotSpotter successfully matched photographs of the same individual 94% of the time. This could be increased to almost 100% by looking at the top 10 picture matches by eye to validate the matching. Additionally, individual spot patterns were unique and stable across years. Using pictures of ventral spots is an easy way to identify individuals, avoids potential rare infection or mortality, and is inexpensive relative to PIT tags and genotyping.

## Graphical abstract

This study evaluates multilocus genotypes and natural markings (e.g., ventral spot patterns) on Texas horned lizards as methods for identifying individuals. Genotyping had high power to identify individuals. Additionally, using HotSpotter software resulted in a 94% success rate in photo matching, which could be further improved with manual validation. Photographic identification proved to be a cost‐effective, reliable, and non‐invasive alternative to traditional tagging methods.

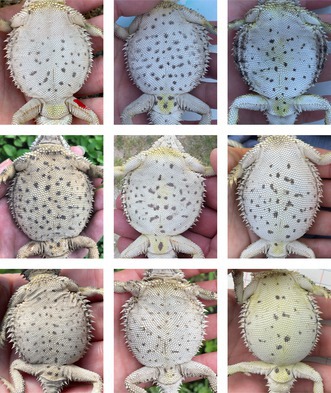

## 1 Introduction

Identifying individuals within a species is vital for monitoring population dynamics and determining appropriate conservation efforts. Traditional methods for marking individual lizards include toe‐clipping, branding, tattooing, and passive integrated transponder (PIT) tags (Plummer and Ferner 2012). However, some of these methods can potentially cause stress, affect performance and survival, and raise concerns about the ethical treatment of animals (Perry et al. 2011). The alternative use of natural markings has proven to be a successful and cost‐effective technique to distinguish between individuals for a wide range of amphibian and reptile species (Sacchi et al. 2007; Sacchi et al. 2010; Sreekar et al. 2013; Morrison et al. 2016; Treilibs et al. 2016; Bauwens et al. 2018; Renet et al. 2019; Tomke and Kellner 2020; Patel and Das 2020; Dunbar et al. 2021; Burgstaller et al. 2021). In addition, the use of multilocus genotypes to discern individuals and detect recaptures of the same individual through non‐invasive sampling (e.g., scat, shed skin) has been used in reptiles but much less often compared with mammals (Bauwens et al. 2018; Tomke and Kellner 2020; Manning et al. 2022).

Texas horned lizards ( *Phrynosoma cornutum* ) occur over a wide range, including northern Mexico, Texas, Oklahoma, Kansas, New Mexico, and parts of Arizona and Colorado (Price 1990). They have experienced noticeable declines in the eastern part of their range and are now considered threatened in Texas and a species of special concern in Oklahoma (Donaldson et al. 1994). Population monitoring of this species requires the use of individually marked lizards. Both toe clipping and PIT tags have been used to individually identify Texas horned lizards (e.g., Henke 2003; Endriss et al. 2007; Burrow et al. 2002; Wolf et al. 2013; this study). Although several studies suggest toe clipping does not negatively affect lizards, a few species experience declines in climbing performance and running speed (Perry et al. 2011). However, this is less of a concern in Texas horned lizards, which rely on crypsis and remain motionless on the ground most of the time to avoid predation and when foraging. In addition, other studies also suggest that PIT tags do not negatively affect growth, blood cell counts, or behavior in Texas horned lizards (Camper and Dixon 1988; Henke 2008).

**FIGURE 1:**
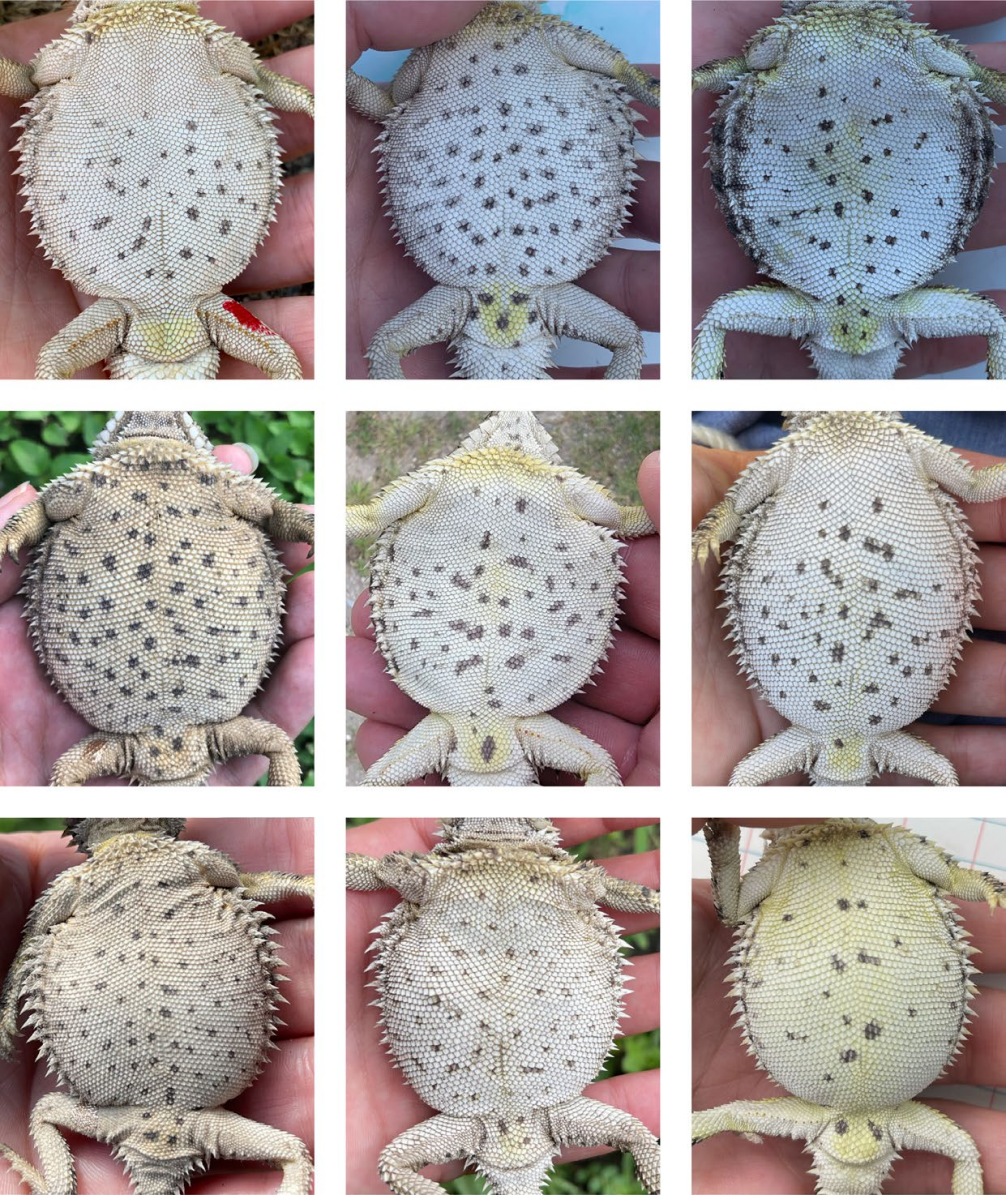
Variability in ventral spot patterns for nine Texas horned lizards. The red spot is a drop of fingernail polish used to identify recaptures within a season.

Ventral markings (i.e., spots) on Texas horned lizards could represent a new approach to identifying individuals (Figure 1). Photographic records of these markings could provide a simpler and more cost‐effective way to track individuals over time. Although it is necessary to capture them by hand to photograph the spots, it would still minimize potential injury from PIT tagging or toe clipping and ethical concerns about the treatment of animals. To determine the effectiveness of ventral spot patterns in identifying individuals, we used data from a long‐term study conducted in south Texas, where individuals were routinely identified using PIT tags and multilocus genotypes (Ackel 2016).

In this study, we determine our power to differentiate individuals based on their multilocus genotypes and then use PIT‐tagged individuals to determine (1) the error rate of our genotyping and (2) how often different individuals have matching genotypes. We then use HotSpotter software to evaluate the distinctiveness of individual ventral spot patterns and their stability across years using a set of individuals identified by PIT tags and/or genotypes (Crall et al. 2013). We chose HotSpotter because it requires minimal processing of pictures before analysis, and it has performed well in previous studies of spot patterns. A recent software comparison study by Burgstaller et al. (2021) found that HotSpotter had the highest precision in green toad individual identification. Similarly, Nipko et al. (2020) compared HotSpotter and Wild‐ID, finding superior performance for HotSpotter in jaguars and ocelots. Other studies on hawksbill sea turtles (Dunbar et al. 2021) and leopard cats (Parks et al. 2019) found nearly 100% matching accuracy with HotSpotter.

## 2 Materials and Methods

### 2.1 Study Area

**FIGURE 2:**
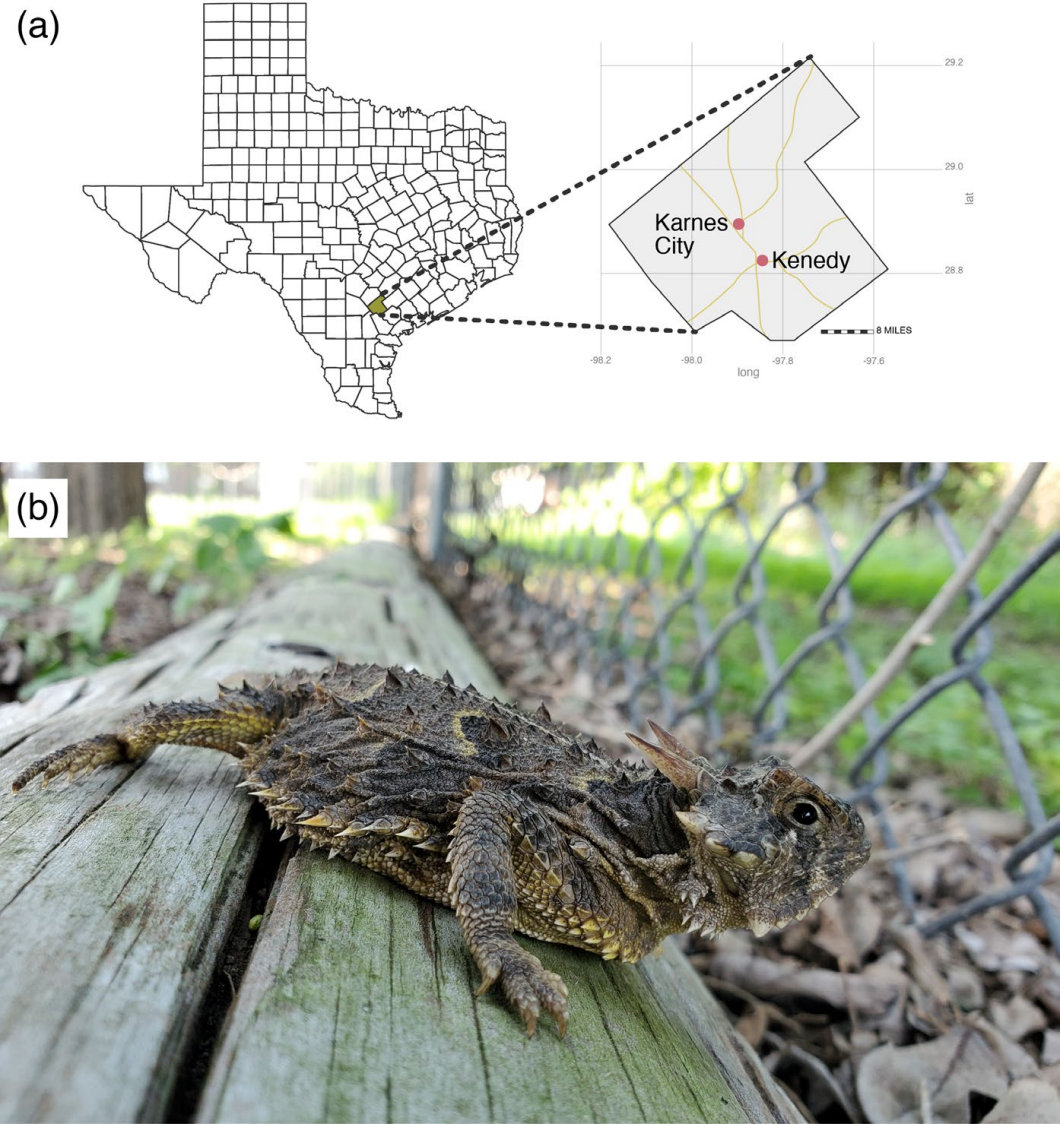
(a) Map of Texas showing the location of Karnes County and the towns of Kenedy and Karnes City. (b) Texas horned lizard in an elementary school playground in Karnes City, Texas. Photograph by D.A. Williams.

Our study was conducted in the towns of Kenedy and Karnes City, Texas, as part of a long‐term study of these populations that was aimed at determining how this species can persist in a human‐altered environment (Ackel 2016; Alenius 2018; Mirkin et al. 2021; Tucker et al. 2023; Wall et al. 2024; Figure 2). Between 2013 and 2021, we surveyed 16 sites for Texas horned lizards from May to August, 8–10 times yearly (Tucker et al. 2023). Lizards were captured by hand, and each year, a DNA sample was obtained from all lizards by swabbing the cloaca with a small Puritan cotton‐tipped applicator (Williams et al. 2012). From 2014 to 2018, in addition to the cloaca swab, lizards > 35 mm snout‐vent length were injected with a BIOMARK HPT8 8.4x1.4 mm PIT tag along their left side and under the skin above the ribs; the injection site was then covered with a drop of surgical glue. We took ventral spot pictures of individuals from 2015 to 2021. Individuals were also marked with a drop of red fingernail polish on the underside of one of the hind legs as a temporary mark indicating that the individual was captured within a field season (Figure 1, top left).

### 2.2 Individual Identification Using Genotypes

DNA was extracted from cloacal swabs following Williams et al. (2012). We genotyped individuals at 10 microsatellite loci and scored the genotypes using GeneMapper 5.0 (Life Technologies), as described in Wall et al. (2024). We calculated genetic diversity indices (i.e., number of alleles, observed and expected heterozygosity), the probability of identity (PI), probability of identity for siblings (PIsibs), and genotype matching using GenAlEx v.6.5 (Peakall and Smouse 2006, 2012). The PI is the probability that two unrelated members of a population will have identical multilocus genotypes, and PIsibs is the probability that two first‐order relatives will have identical multilocus genotypes (Taberlet and Luikart 1999; Waits et al. 2001). The PI is expected to be biased for samples that contain relatives or exhibit population substructure (Waits et al. 2001). We, therefore, calculated PI separately for the two towns and the three regions (east, west, and south) within Karnes City since their populations are significantly differentiated (Wall et al. 2024).

We calculated PI and PIsibs for Karnes City (*n* = 177 individuals used in Wall et al. 2024) and Kenedy (*n* = 41 individuals used in this study) for a total of 218 individuals collected from 2013 to 2015. Of the 177 Karnes City individuals, eight were missing one locus, and three were missing two loci. Two individuals were missing one locus in the Kenedy dataset.

We used a set of 467 individuals with unique PIT tags sampled from 2014 to 2018 (38 from Kenedy, 355 from Karnes City East, 63 from Karnes City West, and 11 from Karnes City South) to validate our genotyping. Of these individuals, 442 were genotyped at all 10 loci, 21 were missing one locus, and four were missing two loci. We used genotypes from all 467 individuals in the year they were captured to estimate our observed PI as the proportion of all pairwise comparisons with identical genotypes. We used a subset of 76 individuals who were recaptured 1–3 times within and between years for a total of 93 recaptures and were resampled for DNA to determine our mean error rate per allele and mean error rate per multilocus genotype (Pompanon et al. 2005).

### 2.3 Individual Identification Using Ventral Spots

Lizards were placed ventral side up in the palm of one hand and photographed using a cell phone camera. We used a damp cloth to wipe the underside of the lizard in cases where dirt or mud might potentially obscure the spot patterns, a common occurrence post‐precipitation. We used a set of 277 ventral photographs taken from 2015 to 2021, comprised of: all individuals captured between 2018 and 2021, as well as those individuals with recaptures one, two, and three years apart between 2015 and 2019 for a total of 224 individuals (Table 1). Of those 224 individuals, 74 were identified by their PIT tags and genotypes, and 150 were identified by their genotypes.

**TABLE 1:** Number of individual Texas horned lizards ( *Phrynosoma cornutum* ) recaptured and photographed.

Recaptures	Individuals
0 years apart (recapture same year)	19
1 year apart (2 consecutive years)	12
2 years apart (3 consecutive years)	5
3 years apart (4 consecutive years)	1
Total	37

### 2.4 Individual Identification With HotSpotter

**FIGURE 3:**
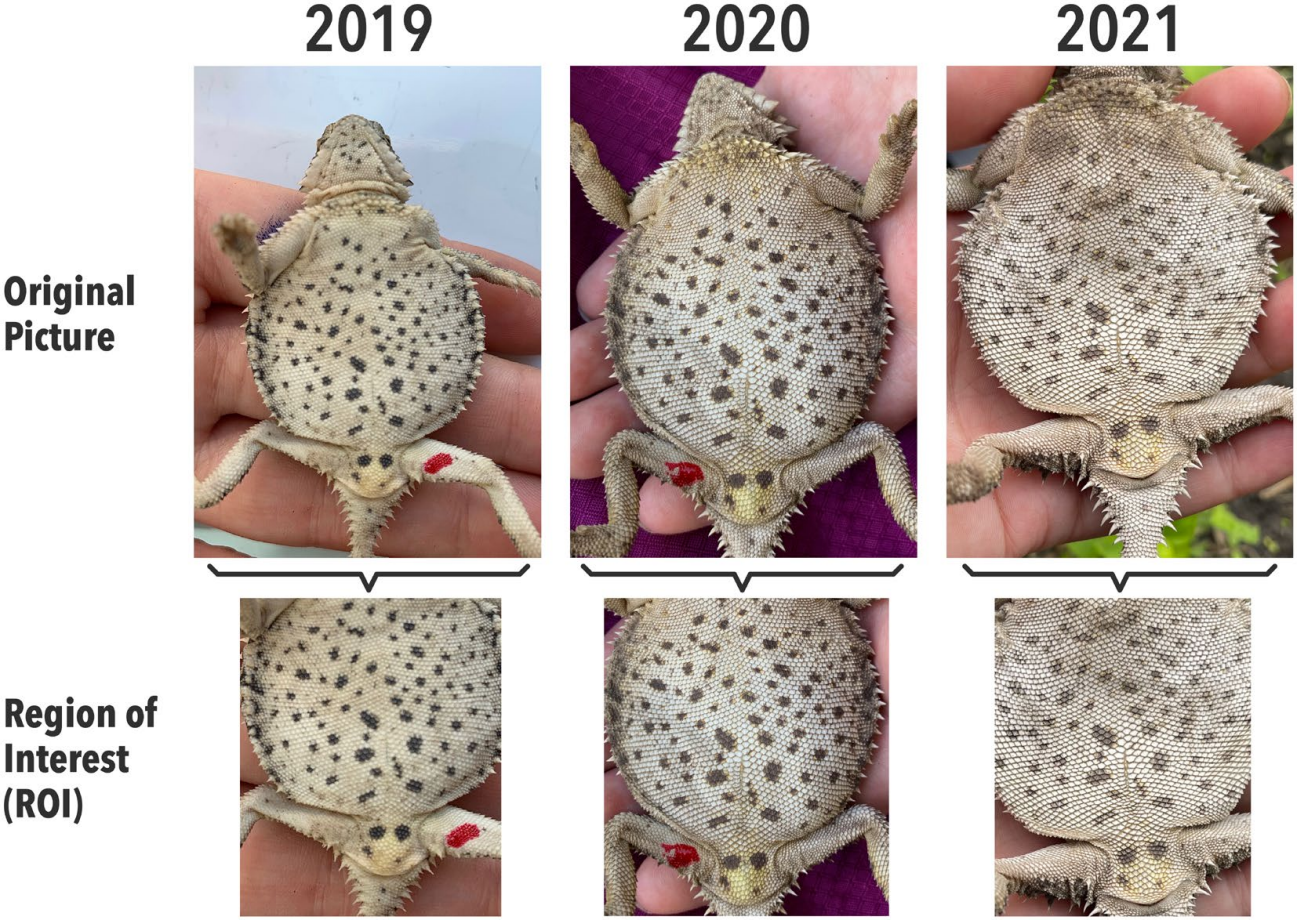
Example of a Texas horned lizard photographed in three consecutive years and the region of interest (ROI) used for identification using HotSpotter. The red spot is a drop of fingernail polish used to identify recaptures within a season.

Before importing the images to HotSpotter, we classified them as reference (*n* = 37), test (*n* = 53), or unique (*n* = 187) based on PIT tags and/or genotyping. *Reference* images represented the initial capture of each individual that was also caught later in the same year or in subsequent years. *Test* images were photos of these subsequent recaptures. *Unique* images were of individuals who were only captured once. After importing all the images to HotSpotter, we selected the region of interest (ROI) by “chipping” the ventral zone of each individual. Specifically, chipping involves clicking on a point on the top left (i.e., the posterior insertion point of the foreleg) and the bottom right side (i.e., below the cloaca) of the lizard to crop the image, creating the ROI (Figure 3).

**FIGURE 4:**
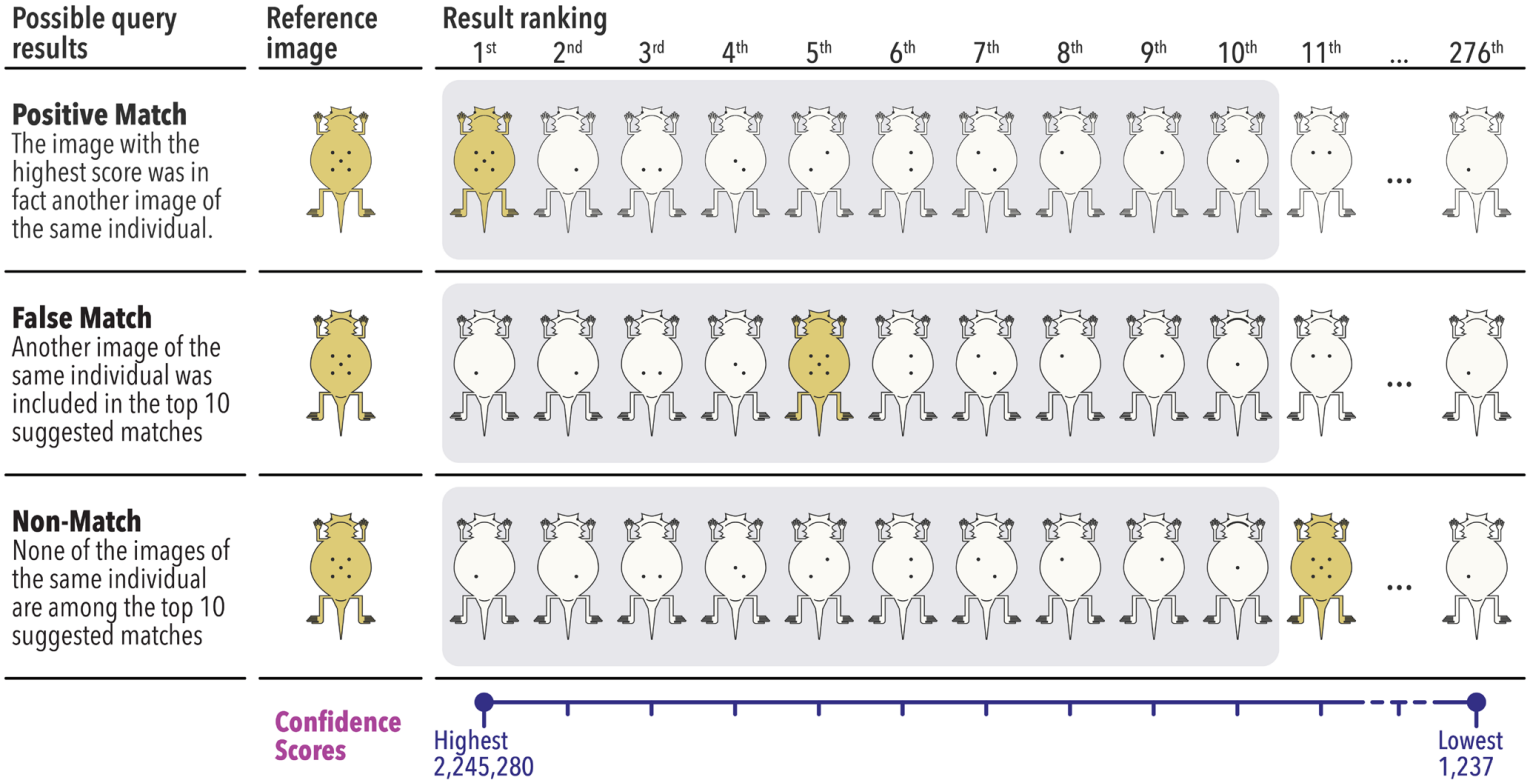
Possible outcomes for queries ran on matching (Reference + Test) images on HotSpotter. For each query run, HotSpotter generates a similarity (confidence) score and ranks the images from most to least similar. The gray area indicates the top 10 matches.

After conducting a query for an image, HotSpotter produced a rank table with confidence scores for all the pictures in the dataset, with higher confidence scores indicating a higher probability of a match. Query results were classified after Nipko et al. (2020) as either positive match, false match, or non‐match (Figure 4). A *positive match* occurs when the image with the highest score is the same individual used in the query. A *false match* is when the image of the same individual is located among the top 10 suggested matches but is not the top‐ranked image. *A non‐match* happens when none of the images of the same individual are in the top 10 suggested matches. We also estimated the false rejection rate (FRR), which is the probability of failing to match two images of the same individual (Jain 2007). This was calculated as the number of reference photographs that did not match the top‐ranked photograph over the total number of true matching images (the 53 *test* photographs). The software did not select the red fingernail polish spots as “hotspots” to compare images. Thus, their presence on some images did not affect the query results.

## 3 Results

### 3.1 Genotype Matching

Karnes City had an average of 8.3 alleles across all three regions of town, and Kenedy had an average of 6.5 alleles per locus (Table 2). The probability that two unrelated individuals (i.e., PI) would match at all 10 loci ranged from 7.3 x 10^−13^ to 3.3 x 10^−9^ in Karnes City and was 1.7 x 10^−09^ in Kenedy (Table 2). The probability that two first‐order relatives would match at all 10 loci (i.e., PIsibs) ranged from 4.1 x 10^−5^ to 3.7 x 10^−4^ in Karnes City and was 3.2 x 10^−4^ in Kenedy (Table 2).

**TABLE 2:** Genetic diversity indices and probability of identity for Texas horned lizard individuals genotyped at 10 microsatellite loci in the towns of Karnes City (KC) and Kenedy (Ken), Texas. Genetic diversity indices are all average ± SE. N is the number of individuals, Na is the number of alleles per locus, HO is observed heterozygosity, HE is expected heterozygosity, PI is the probability of identity, and PIsibs is the probability of identity for siblings.

Town	Pop	N	Na	H_O_	H_E_	PI	PIsibs
KC	East	119	10.9 ± 1.63	0.78 ± 0.01	0.81 ± 0.02	7.3 x 10^−13^	4.1 x 10^−5^
KC	West	42	8.6 ± 0.82	0.80 ± 0.02	0.80 ± 0.02	8.9 x 10^−13^	4.3 x 10^−5^
KC	South	16	5.4 ± 0.62	0.68 ± 0.07	0.66 ± 0.05	3.3 x 10^−9^	3.7 x 10^−4^
Ken	—	41	6.5 ± 0.89	0.67 ± 0.05	0.68 ± 0.04	3.5 x 10^−9^	4.1 x 10^−4^

Three recaptures mismatched at one allele each from the original capture (at three different loci), resulting in a mean error rate per allele of 0.0016 (3 of 1860 alleles across the 93 recaptures) and a mean error rate per multilocus genotype of 0.032 (3 genotypes of 93 recaptures). Of the 467 individuals with unique PIT tags, two pairs matched at 9 and 10 loci in Karnes City East, and one at nine loci in Kenedy. These genotypes were confirmed through reamplification and were all male–female pairs captured at the same site within the same year or in adjacent years. The observed PI values for these three matches were closer to the PIsibs estimate (Table 3). Of the remaining matches, three pairs matched at eight loci, six at 7 loci, 24 at six loci, 117 at five loci, and 553 at four loci.

**TABLE 3:** Comparison of observed PI values (ObsPI) to the theoretical PI and PIsibs values in the towns of Karnes City (KC) and Kenedy (Ken), Texas for pairs with identical and close genotype matches. N loci is the number of matching loci between pairs; ObsPI is calculated as the observed proportion of all possible pairs of individuals with identical multilocus genotypes; PI is the theoretical probability of identity; PIsibs is the theoretical probability of identity for siblings.

Population	N loci	ObsPI	PI	PIsibs
KC East	10	1.6 x 10^−5^	7.3 x 10^−13^	4.1 x 10^−5^
KC East	9	1.6 x 10^−5^	7.9 x 10^−12^	1.0 x 10^−4^
Ken	9	1.4 x 10^−3^	1.7 x 10^−8^	7.9 x 10^−4^

### 3.2 HotSpotter Image Matching

**FIGURE 5:**
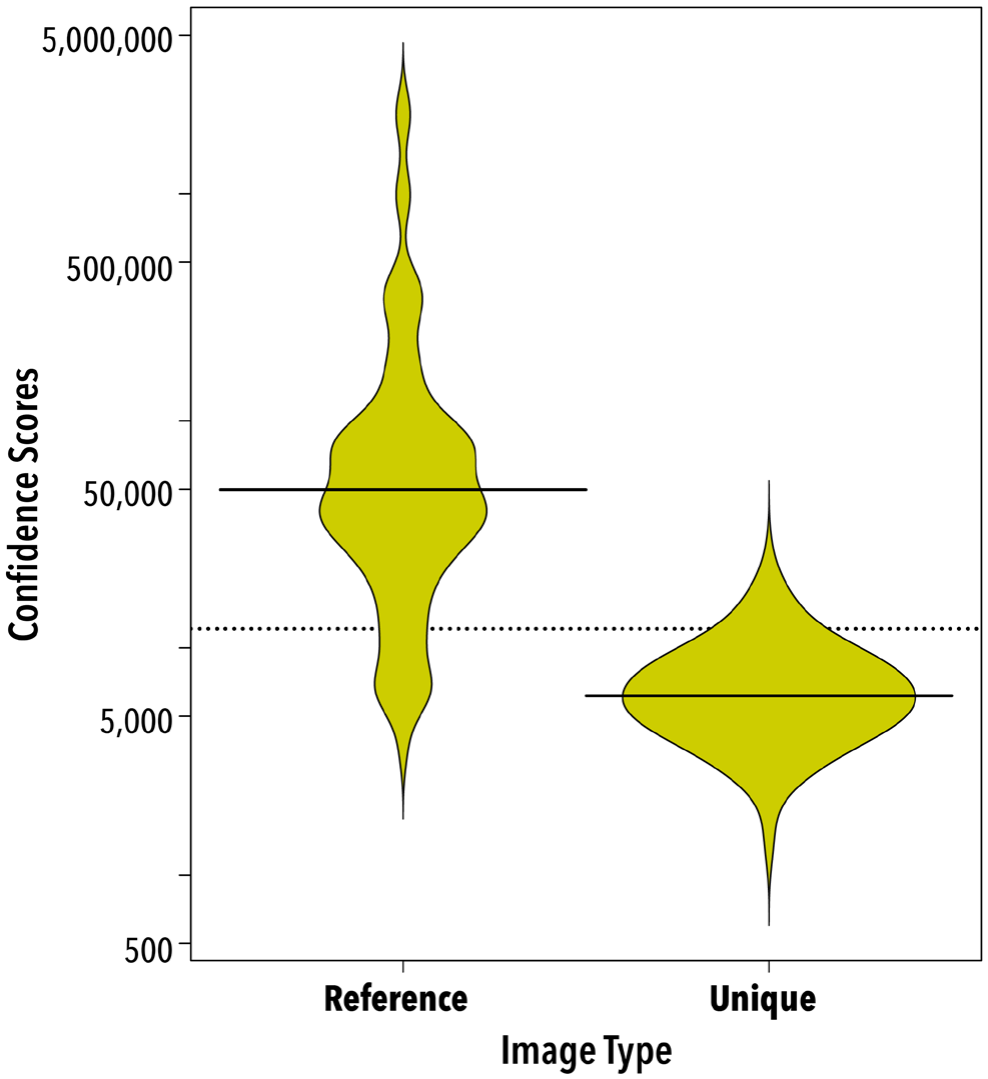
Beanplots depicting the confidence scores of matching images (Reference + Test; *n* = 90) and non‐matching images (Unique; *n* = 187) of Texas horned lizards generated by HotSpotter. Each “bean” shows the distribution of scores and the black lines in each bean indicate the average. The y‐axis is log transformed. The dotted line indicates the overall average score.

HotSpotter scores were higher for images of the same individual than for images of individuals only captured a single time (277 images; Figure 5). The recapture confidence scores ranged from 3644 to 2,245,280 with a mean value of 140,939, and the confidence scores of photographs without recaptures ranged from 1237 to 26,409 with a mean value of 6949 (Figure 5). Of the matching scores, 26.7% (*n* = 24) overlapped with the non‐matching scores (Figure 5). The success rate of a positive match was 94.4% (85 pictures of 90), the false match rate was 4.4% (four pictures), and the non‐match rate was 1.1% (one picture). These five mismatches result in an FRR of 0.094. It was easy to visually determine the correct match in the top ten photographs in all the false matches. Of the four false matches, two pictures had faint spots, and one lizard was photographed at a slight angle. There were no apparent reasons for the mismatch in the fourth picture; all images of that individual were clear, and visual inspection found identical spot patterns. The lone non‐match was probably caused by some soil specks on the underside of the individual in one of the pictures. Spot patterns were also a good identification check for the pairs with identical and near‐identical genotypes presented above. In all cases, the spot patterns did not match between the individuals.

## 4 Discussion

This study was in the unique position of possessing a dataset of individuals that were PIT tagged, genotyped, and photographed, which allowed us to validate our genotyping and natural markings for individual identification. Both towns have significantly lower genetic diversity than populations living in more natural areas, and there is short‐distance dispersal due to isolation by distance and urbanization (Wall et al. 2024). Nevertheless, we still realized high power to differentiate individuals in these towns using genotyping; our genotyping error rate was low and within the range reported in other studies (Pompanon et al. 2005). This set of loci will have even more power to identify individuals in more diverse populations living in natural areas. The probability of identity can be biased by population substructure, sampling relatives, or past demographic events that disrupt Hardy–Weinberg equilibrium (Waits et al. 2001). We could control for substructure by calculating PI separately for the towns and regions in Karnes City. However, given the short‐distance dispersal in these towns, we probably included some closely related individuals in our calculations. There is also evidence of past genetic bottlenecks in both towns, which may have impacted PI (Wall et al. 2024). Given their temporal and spatial proximity, the three pairs of individuals that matched at nine and 10 loci may have been full siblings. Our observed PI values for Karnes City East and Kenedy were closer to the PIsibs values that have been observed in other studies, leading to the recommendation that PIsibs is probably a better, more conservative estimate of PI in many natural populations (Waits et al. 2001).

Natural ventral markings in Texas horned lizards are unique among individuals and stable across years. In this study, the success rate of a positive match was 94% using HotSpotter. This positive match rate is similar to previous studies (Nipko et al. 2020; Burgstaller et al. 2021). Our FRR was also within the range commonly reported for other animals (Bolger et al. 2012). The positive match rate could be increased to almost 100% by looking at the top 10 pictures by eye to validate the matching. In addition, we found it easy to use a catalog of images organized by site to identify individuals by eye in the field within a year and even between consecutive years. The use of image‐matching software, however, is better suited for searching large numbers of individuals across multiple years. The only non‐match we found highlights the need to clean dirt from the underside of the lizard to remove false spots or uncover spots that may be obscured by soil. We also found that spot patterns were useful for evaluating close genotype matches for potential identification errors.

Using pictures of ventral spots is an easy way to identify individuals and avoid rare but potential infections or mortality. Texas horned lizards often remain motionless when approaching them, so it is easy to capture them by hand to obtain photographs. We do not know how stressful capturing or handling is for this species, although several studies suggest handling has minimal effects on stress levels in lizards. Langkilde and Shine (2006) measured plasma corticosterone levels in a scincid lizard (*Eulamprus heatwave*) and found that handling and toe clipping appear to be significantly less stressful procedures than PIT tagging or holding individuals in unfamiliar enclosures. Another study that measured blood lactate concentration and heart rate found that hand‐capturing iguanas ( *Cyclura carinata* ) was significantly less stressful than using a noose pole to capture individuals (Colosimo et al. 2024). Several studies have successfully used pictures of free‐ranging lizards to identify individuals, including the Slater's skink (*Liopholis slateri*; Treilibs et al. 2016) and the prairie lizard ( *Sceloporus consobrinus* ; Tomke and Kellner 2020). Texas horned lizards have complex dorsal color patterns and protuberances (Rhoads and Williams 2023; pers. obs.) and, if found to be a reliable individual identification method, would probably present more challenges to quantify than the ventral spot patterns. The advantage, however, would be that pictures of individuals' dorsums could be taken without capturing them.

Using natural ventral markings to identify individuals is also inexpensive since pictures can be obtained using a cell phone, and HotSpotter is freeware. Toe clipping is limited when there is a need to mark large numbers of individuals with unique combinations, and PIT tags can be expensive for large numbers of individuals (~ USD 5.00 per individual). Genotyping using DNA extracted from toe clips or cloacal swabs can effectively identify individuals and can provide estimates of genetic diversity, effective population sizes, and dispersal. Nevertheless, DNA methods require expensive specialized laboratory equipment and expertise and can also be costly for monitoring large numbers of individuals (~ USD 6.00 per individual in our laboratory). If these genetic data are unnecessary, photographing spot patterns is a more straightforward way to identify individuals. However, recently, we have encountered individuals in central Texas that lack ventral spots or have very few light spots, making it necessary to use alternative methods to identify these individuals in the long term (pers. obs.).

## Author Contributions

**Daniella Biffi:** conceptualization (Equal), Data curation (Equal), Formal analysis (Equal), Investigation (Equal), Methodology (Equal), Project administration (Equal), Visualization (Equal), Writing – original draft (Equal), Writing – review and editing (Equal). **Mary R. Tucker:** data curation (Equal), Formal analysis (Equal), Investigation (Equal), Methodology (Equal), Visualization (Equal), Writing – original draft (Equal), Writing – review and editing (Equal). **Alexis Ackel:** formal analysis (Equal), Investigation (Equal), Methodology (Equal), Writing – review and editing (Equal). **Dean A. Williams:** conceptualization (Equal), Data curation (Equal), Formal analysis (Equal), Funding acquisition (Lead), Investigation (Equal), Methodology (Equal), Writing – original draft (Equal), Writing – review and editing (Equal).

## Ethics Statement

Field activities were approved by Texas Christian University Institutional Animal Care and Use Committee (IACUC) protocols 13–05, 15–10, 18–05, 2021–04, and Texas Parks and Wildlife Scientific Research Permit No. SPR‐0613‐073.

## Conflicts of Interest

The authors declare no conflicts of interest.

## Data Availability

Data for this study are available at: https://doi.org/10.18776/tcu/data/66887.
